# Effect of Solution Process on Microstructure and Properties of Cr-Mn-N Austenitic Stainless Steel

**DOI:** 10.3390/ma18061290

**Published:** 2025-03-14

**Authors:** Xianbang Dong, Fuxing Wang, Lei Huang, Jian Lan, Yuzhi Chen, Bingji Li, Hao Yu

**Affiliations:** 1 School of Materials Science and Engineering, University of Science and Technology Beijing, No. 30 Xueyuan Road, Haidian District, Beijing 100083, China; d202210209@xs.ustb.edu.cn (X.D.); m202220477@xs.ustb.edu.cn (F.W.); 2 Guangxi BG New Materials Co., Ltd., Beihai 536000, China; hleybgxc@foxmail.com (L.H.); 18277990135@163.com (J.L.); fish-tgkw881214@163.com (Y.C.); jige172010016@163.com (B.L.)

**Keywords:** solid solution, Cr−Mn−N austenitic stainless steel, grain size, precipitated phases, strengthening mechanism

## Abstract

To elucidate the impact of the solid solution process on the microstructure and mechanical properties of Cr−Mn−N austenitic stainless steel, comparative experiments were conducted with varying solid solution temperatures and durations. The results indicate that the grain size gradually increases with increasing solid solution temperature and duration. When the temperature reaches a high level (1120 °C) or is maintained at 1080 °C for an extended period (25 min), the smaller grains are progressively engulfed by the adjacent larger grains, resulting in a swift augmentation in grain size and heterogeneity. In the hot rolled specimens, a considerable quantity of precipitates with large sizes (200 nm) is observed. After the solid solution treatment, the precipitate dimensions are significantly diminished, and their volume fraction is significantly influenced by the temperature of the solid solution. EDS and HRTEM were used to determine that the main precipitated phases after hot rolling and solid solution treatment were Cr_7_C_3_, Cr_23_C_6_ and Cr_2_N. With the increase in the solid solution temperature and time, the increment of grain boundary strengthening and dislocation strengthening decreases, while the contribution of precipitation strengthening initially increases before subsequently decreasing, which is the reason why the experimental steels with solid solution temperature of 1040 °C and solid solution temperature of 1080 °C held at 5 min still have the same mechanical properties despite the difference in solid solution treatment processes.

## 1. Introduction

In recent years, with the gradual scarcity of global nickel resources and price fluctuations, nickel-saving stainless steels, especially 200-series austenitic stainless steels, have received widespread attention. Unlike traditional 300-series austenitic stainless steels, 200-series stainless steels achieve significant cost savings by reducing the nickel content and using manganese and nitrogen instead of nickel to stabilize the austenitic phase [1,2,3,4]. The lower cost of 200-series stainless steels and their ability to retain their austenitic structure at low temperatures make them an economically competitive alternative in many areas. Moreover, 200-series austenitic stainless steels not only offer economic advantages but also retain excellent ductility and machinability at higher strengths [5,6,7]. Especially under cold working conditions, 200-series stainless steels exhibit high yield and tensile strengths [8], making them widely used in a number of fields with high strength requirements.

Precipitation phases in austenitic stainless steel hot rolled sheet have a significant effect on its properties, especially corrosion resistance. Common precipitation phases are chromium carbides and nitrides, such as Cr_23_C_6_ and Cr_2_N [9,10], which usually precipitate along grain boundaries, leading to the formation of chromium-poor zones, which significantly increase the intergranular corrosion susceptibility of the material [11,12,13,14]. Intergranular corrosion is particularly serious in corrosive media and may lead to early failure of stainless steel when subjected to external forces [15].

In order to optimize the properties of austenitic stainless steels, it is crucial to control the formation of precipitated phases and the solid solution process. Effective control of the degree of the dissolution of the precipitated phases through a suitable solution annealing process can optimize grain size and mechanical properties [16,17]. Under ideal solid solution treatment conditions, the precipitated phases can be re-dissolved into the matrix, improving the homogeneity and corrosion resistance of the material, obtaining a reasonable grain size, and improving the toughness and ductility of the material [18,19]. Inappropriate solid solution treatment conditions may result in abnormal grain growth and stress concentrations, thereby decreasing the material’s crack resistance during use.

Current research on 200-series austenitic stainless steels tends to focus on experimental results demonstrating performance enhancement, and although precipitation phases, grain boundaries, etc., are known to contribute to the mechanical properties of 200-series austenitic stainless steels [20,21,22,23,24,25], the interactions of the strengthening mechanisms and the dominant factors are still understudied. Therefore, this paper, through the setting of different solution annealing temperatures and times, focuses on a newly developed 200-series austenitic stainless steel hot rolled sheet, systematically studying the impact of solid solution annealing process parameters on precipitation phase redissolution and grain growth and exploring its strengthening mechanism to determine the optimal process. The goal is to maximize stainless steel’s comprehensive performance, ensuring its reliability and safety in various application scenarios. The comprehensive performance of stainless steel ensures its reliability and safety in various application scenarios and provides important theoretical support and a practical basis for the practical application of 200-series austenitic stainless steel.

## 2. Materials and Methods

### 2.1. Experimental Materials and Processes

**Figure 1:**
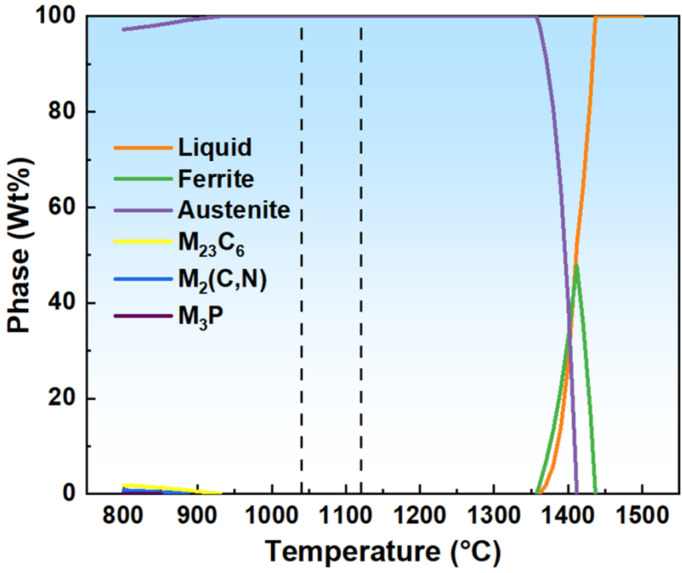
Phase transition curve during solidification of experimental steel. (The area inside the dotted line is the final solution temperature range).

The newly developed 200-series austenitic stainless steel, which is the subject of this study, offers a cost-effective alternative to traditional stainless steel due to its reduced nickel content. This material has been increasingly utilized in various applications, including construction, decoration and other fields. The chemical composition of this steel, as detailed in Table 1, typically includes higher manganese and nitrogen levels to compensate for the lower nickel content, ensuring a balance of corrosion resistance and mechanical strength. Thus, 200-series austenitic stainless steel, containing a large number of N, Mn and other austenite−forming elements, is also austenitic at room temperature organization but, after hot rolling, will precipitate a certain amount of carbide and nitride; in addition to the grain size, the organization will also change due to processing [9,17]. The purpose of solid solution annealing of this steel is to dissolve the precipitated carbon and nitrides in austenite at elevated temperatures while maintaining the solidified austenite at ambient temperatures using rapid cooling and adjusting the organizational grain size during the annealing process for softening purposes. According to the composition, JMatPro software (Version 7.0.0.) was used to predict the phase transition of the experimental steel during solidification in the range of 800−1500 °C, as shown in Figure 1. Combined with the findings, it was concluded that the precipitation onset temperature of the Cr_23_C_6_ phase of the experimental steel was in the range of 920−960 °C [26,27], and the precipitation onset temperature of Cr_2_N was around 950 °C [28]. Therefore, in order to ensure a good solid solution effect, the solid solution process of each specimen in this study is shown in Table 2.

### 2.2. Experimental Methods

The size of the sample for microstructure observation was 12 mm × 10 mm × 6 mm. After sandpaper grinding and mechanical polishing, it was corroded with aqua regia solution (concentrated nitric acid and concentrated hydrochloric acid 1:3) that was left to stand for 30 min, and the test samples were observed using optical microscope (OM) and scanning electron microscope (SEM) to analyze the microstructural morphology and grain size.

Samples of size 10 mm × 10 mm × 3 mm were analyzed using electron backscatter diffraction (EBSD) (Zeiss ULREA 55-type, Oberkochen, Germany). Before testing, the surfaces of the specimens were ground with sandpaper and mechanically polished. The surface was electrolytically polished with 5 vol% perchloric acid alcohol solution (volume fraction of 5 vol%) at 25 V for 60 s, keeping the current between 1.0 and 1.4 A and the temperature around −20°C. EBSD analysis was carried out using Zeiss ulrea 55 field emission electron microscope (Oberkochen, Germany) and HKL nordys F+ probe (Oxford Instruments, Oxford, UK) with a step size of 1.0 μm. After processing by the AZtecCrystal software(Version 2.1), the grain sizes of the enhanced specimens were quantitatively analyzed, along with grain boundary distribution and recrystallisation. Export the grain size data calculated by EBSD for each sample, plot the grain size distribution histogram including cumulative frequency data for each specimen, and fit the particle distribution using the log-normal method.

The procedure for creating a carbon film extraction replica for the observation of precipitation phases involves the following detailed steps: Initially, the metallographic specimen undergoes grinding, polishing, and etching processes. Subsequently, a thin layer of carbon film, approximately 20 nm in thickness, is applied to the specimen’s surface under vacuum conditions. Following the carbon deposition, a hobby knife is used to delineate a grid pattern with 3 × 3 mm squares on the carbon-coated surface. The specimen is then immersed in a 30% diluted aqua regia solution, allowing for the gradual extraction of the carbon film. Once the film is detached, it is retrieved using a copper mesh. The carbon film is subsequently rinsed in alcohol, spread out in deionized water, and, finally, the copper mesh is positioned on filter paper to facilitate drying. This completes the sample preparation process. The precipitated phase in the prepared carbon film sample was observed and photographed with a Tecnai G2 F20 field emission transmission electron microscope produced by FEI, Hillsboro, OR, USA, and the diffraction spots were obtained and calibrated to determine the composition of the precipitated phase.

A rectangular cross-section tensile specimen, with a thickness of 3 mm, a width of 10 mm, and a marking length of 25 mm, was processed from a heat-treated sheet. According to GB/T228.1-2010 [29] standard, the room temperature tensile test was carried out on the electronic universal testing machine model CMT5205 (MTS Systems Corporation, Eden Prairie, MN, USA), and the average value was obtained from two tests.

## 3. Results

### 3.1. Mechanical Properties and OM Analysis

#### 3.1.1. Mechanical Properties Analysis

Table 3 summarizes the mechanical properties of 200-series austenitic stainless steels under different solid solution treatment conditions, including the specified plastic elongation strength, tensile strength, elongation at break and strength-to-yield ratio. Table 3 shows that the elongation at break of each specimen is stable between 55% and 60%. Specimen #1, with a solid solution temperature of 1040 °C, and specimen #2 at 1080 °C show a high tensile strength and strength−to−yield ratio, and with the increase in temperature to 1120 °C (#5) and the extension of solid solution time to 15 min (#3) and 25 min (#4) at 1080 °C, the strength of the specimens decreases to below 400 MPa, but the elongation at break improves.

#### 3.1.2. OM Microstructure Analysis

**Figure 2:**
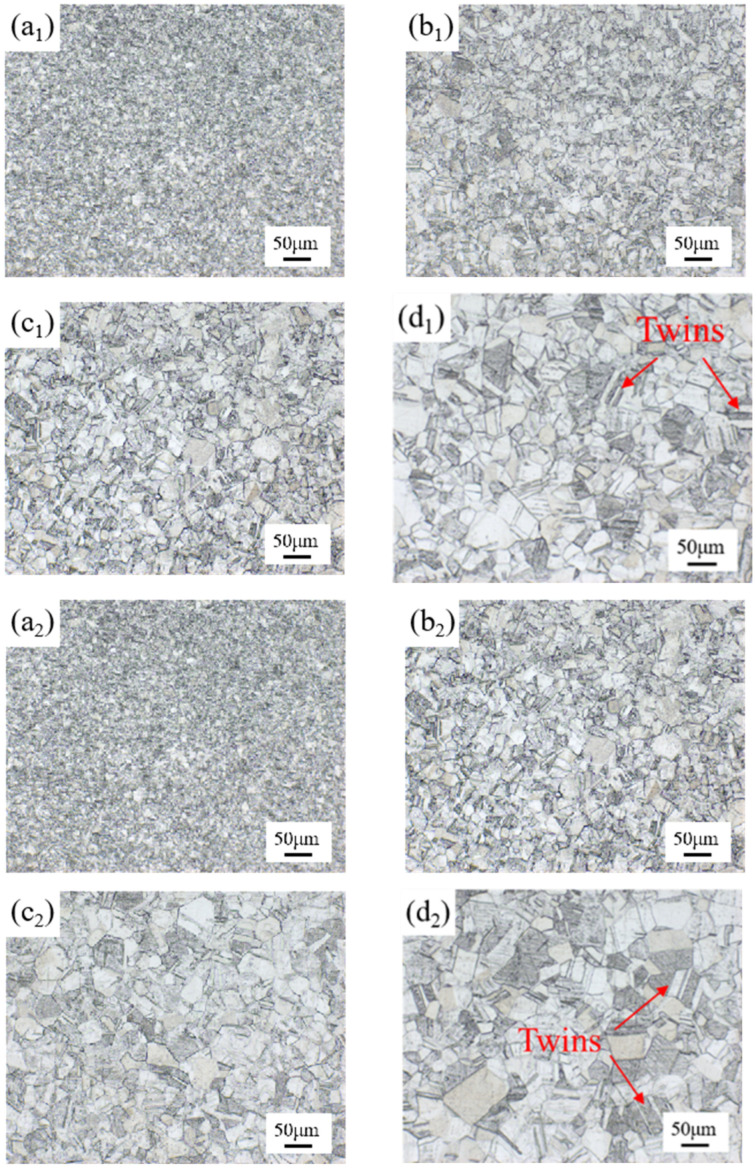
Microstructure of specimen OM. (**a_1_**) Hot rolled specimen; (**b_1_**) 1040 °C, 5min; (**c_1_**) 1080 °C, 5 min; (**d_1_**) 1120 °C, 5 min; (**a_2_**) hot rolled specimen; (**b_2_**) 1080 °C, 5 min; (**c_2_**) 1080 °C, 15 min; (**d_2_**) 1080 °C, 25 min.

Figure 2 demonstrates the OM microstructure of the specimens in the hot rolled state and at different solid solution temperatures (1040 °C, 1080 °C and 1120 °C) and solid solution times (5 min, 15 min and 25 min). It can be seen that the original specimen (a_1_) has fine grains with a high number of grain boundaries. The grain size increased significantly after the solid solution, and many annealed twins appeared. With the increase in the solid solution temperature and time, the grain size increases gradually.

#### 3.1.3. Grain Size Analysis

In order to study the influence of solution temperature and time on the grain size distribution and uniformity of 200-series austenitic stainless steel, the image analysis software Nano Measurer (Version 1.2) was used to analyze the grain size of hot rolled and solution-treated samples. The grain size distribution, average grain diameter and distribution characteristics of each sample are analyzed in detail. Specifically, the grain size distribution, the average diameter of the grains and their distribution characteristics of each specimen were analyzed.

**Figure 3:**
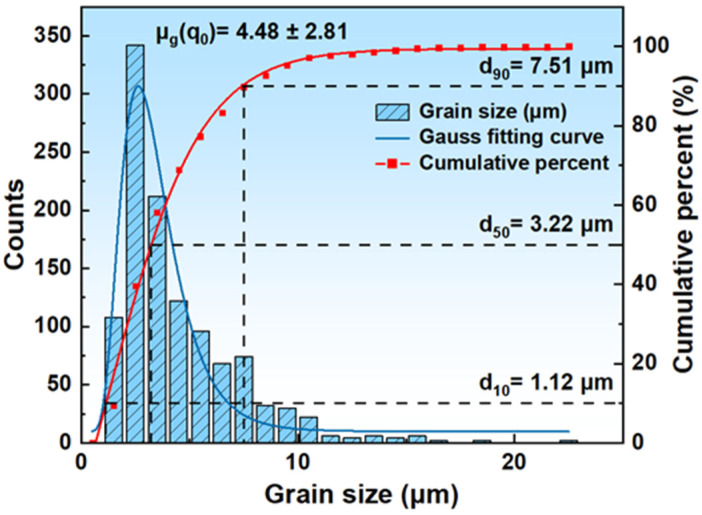
Grain size distribution of the original specimen.

Figure 3 shows the histogram of the data analysis of the grain size distribution of the hot rolled specimens of 200-series austenitic stainless steel after hot rolling, including the cumulative frequency obtained by fitting the log-normal distribution function (log-normal function). Equation (1) is the expression of the log-normal function, where D is the equivalent circle diameter, which is the diameter of a circle with an area equal to a given shape (usually an irregular shape), and E is the standard deviation. The equivalent circle diameter of the original specimen was calculated to be 4.48 ± 2.81 μm, with a large fluctuation in the grain size, indicating that the original specimen was not uniform in grain size. R^2^ = 0.943, showing that the model fits well.

(1) $$\mu_{g}(q_{0})=D\pm E$$

**Figure 4:**
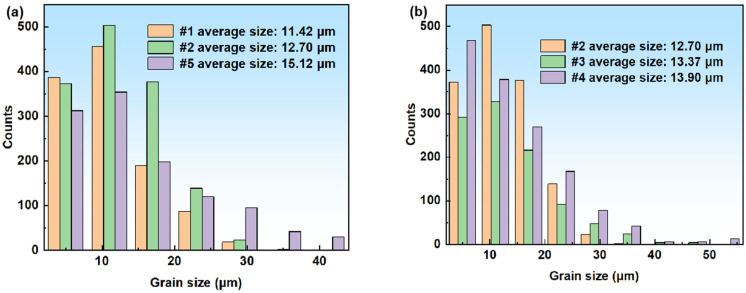
Grain size distribution of the hot rolled specimens in solid solution at different temperatures and times: (**a**) variation with temperature; (**b**) variation with time.

Figure 4 shows the statistical diagram of the grain size of the austenitic stainless steel hot rolled steel plate in the solid solution at different temperatures and times. The average grain size of the sample in the solution at 1040 °C, 1080 °C and 1120 °C for 5 min is 11.42 μm, 12.70 μm and 15.12 μm, respectively. The average grain sizes of the samples were 12.70 μm, 13.37 μm and 13.90 μm in the solution at 1080 °C for 5 min, 15 min and 25 min, respectively. The grain size increases with the increase in solution temperature and time. The grain size of samples #1, #2 and #3 is basically within 30 μm, and the size is relatively uniform, while there are many grains larger than 30 μm in samples #4 and #5.

Through the data analysis histogram of particle size distribution, including cumulative frequency, three percentage characteristic particle sizes of D10, D50 and D90 can be obtained. These particle sizes represent the proportion of grains smaller than this size in the whole grain. The characteristic particle size of each sample was obtained by analyzing the histograms of different solution processes, and the relative ratio of percentage diameter and span ratio were calculated by Formula (2), which are listed in Table 4.

(2) $$R=(d_{nx}-d_{ni})/d_{ni}$$

where R represents the percentage diameter relative ratio under different solution treatment processes; n is the general parameter, in this case, 10, 50, 90; d_ni_ is the characteristic particle size of the three percentages of the hot rolled sample; x is the basic variable, which refers to the sample #1, #2, #3, #4 and #5 under different solution treatments.

**Figure 5:**
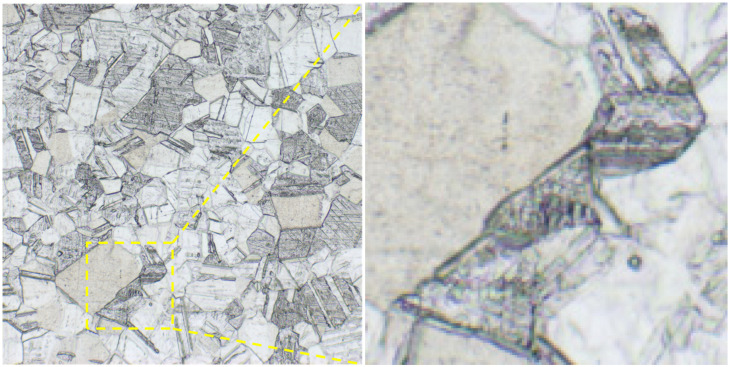
Grain swallowing results with an increase in grain size inhomogeneity.

Comparative analysis of the span ratios under different solid solution treatment processes reveals that the span ratios of specimens #1, #2, and #3 have decreased, and the span ratios of specimens #4 and #5 have increased. According to the abnormal grain growth mechanism, it is known that if there are grains with larger sizes in the initial grains, these relatively larger grains are more likely to grow. Initial grain size generally has parabolic distribution characteristics. Set r for any one of the grain radius, r_c_ for the critical radius, and if r > r_c_, then the grain grows, and vice versa; the grain gradually disappears [30]. Therefore, under higher temperature (1120 °C) and longer time (25 min) conditions, the small grains are gradually subsumed by the surrounding larger grains [31], as shown in Figure 5 This phenomenon can be attributed to the fact that larger grains exhibit lower grain boundary energies, which contributes to their enhanced stability within a specific region. But this effect led to a rapid increase in grain size and an increase in inhomogeneity. The maximum grain size of 85 μm was obtained after measurements.

### 3.2. EBSD Analysis

**Figure 6:**
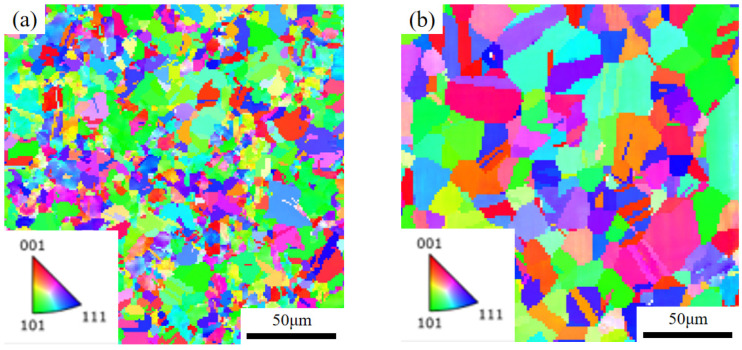

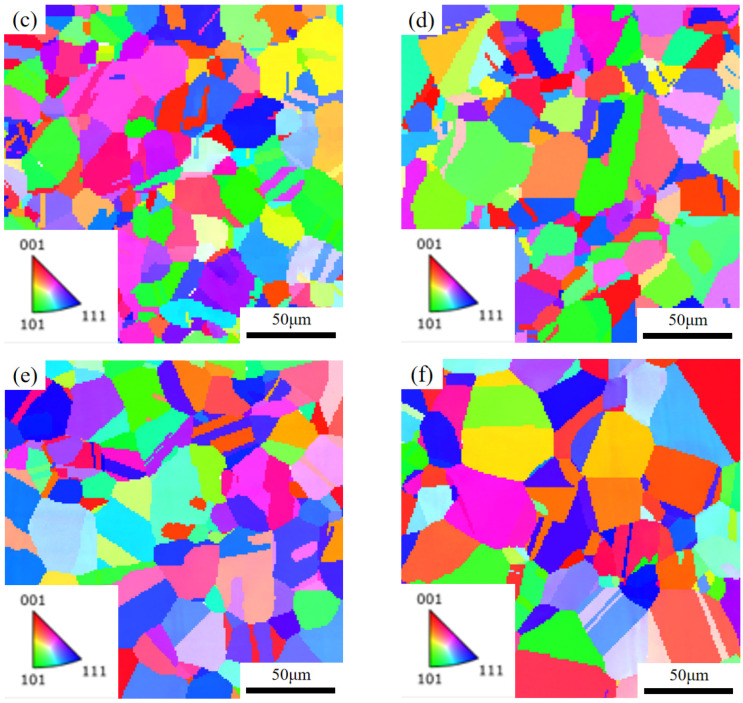
IPF diagrams of (**a**) hot rolled specimen; (**b**) 1040 °C, 5 min; (**c**) 1080 °C, 5 min; (**d**) 1080 °C, 15 min; (**e**) 1080 °C, 25 min; (**f**) 1120 °C, 5 min.

The EBSD technique can also be used to count the grain size of steel. It has been shown that large angular grain boundaries with an orientation difference of more than 15° can split the crystal structure and form so-called ‘equivalent grains’, whose diameter is known as the effective grain size. Figure 6 shows the inverse polefigure (IPF) of each specimen obtained using EBSD. When statistically analyzing the effective grain size of steel, use a 15° misorientation as the criterion for delineation, exclude boundary grains and disregard the influence of twin boundaries in special grain boundaries. According to statistical analysis, the average effective grain size for each specimen, as detailed in Table 5, is determined by considering the distribution and characteristics of grain sizes. The average effective grain size of each specimen is within 0.2 μm of the size measured by the Nano Measurer, which verifies the reliability of the Nano Measurer’s measurement results.

**Figure 7:**
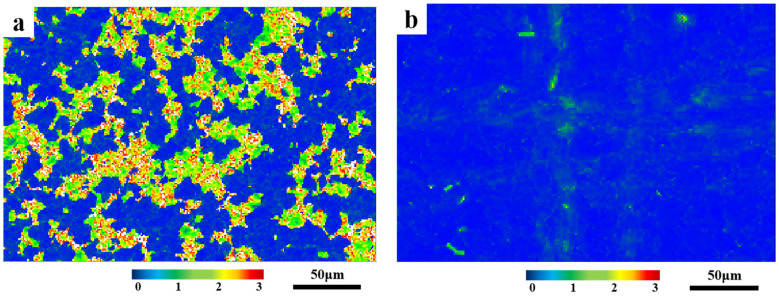

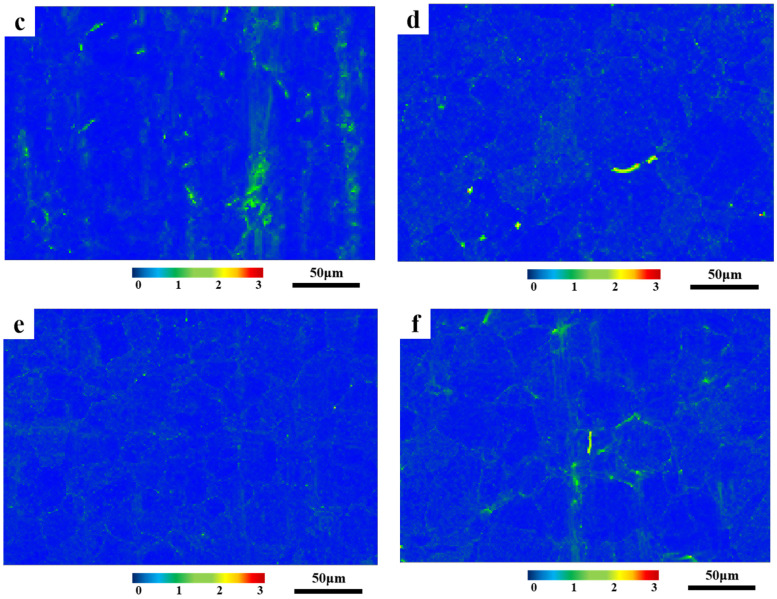
KAM of (**a**) hot rolled specimen; (**b**) 1040 °C, 5 min; (**c**) 1080 °C, 5 min; (**d**) 1080 °C, 15 min; (**e**) 1080 °C, 25 min; (**f**) 1120 °C, 5 min.

After this, the geometric dislocation density of each specimen was quantitatively calculated using KAM plots [5], and the results are shown in Figure 7 and Table 6. It can be seen that the solid solution treatment can effectively reduce the dislocation density of the specimens in the hot rolled state, and the degree of reduction in dislocation density is greater with the increase in solid solution temperature and the prolongation of solid solution time.

### 3.3. Study on Precipitation Behavior Under Different Solid Solution Processes

In 200-series austenitic stainless steels, precipitated phases can seriously affect the mechanical properties and corrosion resistance of the materials. In order to study the morphology and distribution of precipitated phases under different solid solution treatment conditions, this study used transmission electron microscopy (TEM) to observe the distribution and type of precipitated phases in the original specimen and the solid solution-treated specimen and combined with energy spectroscopy (EDS) analysis to determine the chemical composition of the precipitated phases.

**Figure 8:**
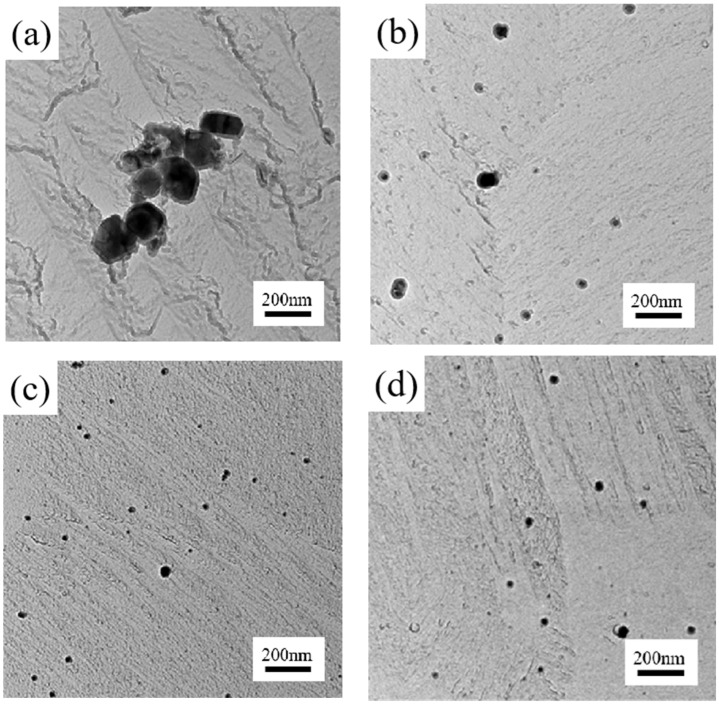

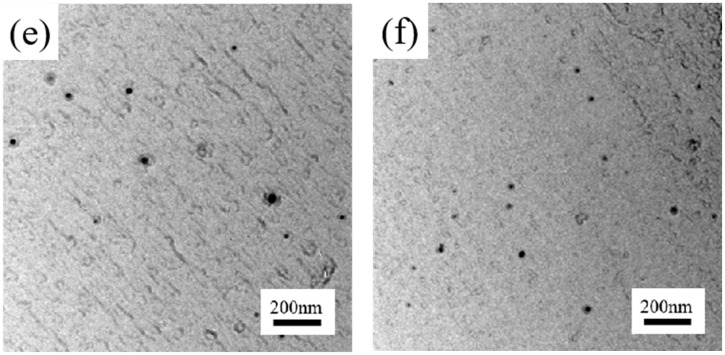
Bright field TEM results of analyzed phases: (**a**) hot rolled specimen; (**b**) 1040 °C, 5 min; (**c**) 1080 °C, 5 min; (**d**) 1080 °C, 15 min; (**e**) 1080 °C, 25 min; (**f**) 1120 °C, 5 min.

Figure 8 shows the bright field TEM results of the precipitated phases in the hot rolled specimen and the carbon film extracted replica samples of different solid solution-treated test steels. From the TEM results, it can be found that there are more precipitated phases in the original specimen and the size is larger, most of the precipitated phases are more than 200 nm in size, and, at the same time, there are many precipitation aggregation areas, which affect the mechanical properties and corrosion resistance of stainless steel hot rolled sheet. After solid solution treatment, the precipitated phase distribution is uniform, and the size is obviously smaller. Only sample #1 has several precipitated phases with a size larger than 60 nm, and the other four samples of the precipitated phase size are below 60 nm. The average size of the precipitated phase particles, the density of the precipitated phase and the volume fraction of the precipitated phase were statistically analyzed. The formula for calculating the volume fraction of the precipitated phase is as follows [32].

(3) $$f=\frac{\frac{\pi }{6}\sum_{n=i}^{n}d_{i}^{3}}{A(t_{V}+\bar{d})}$$

where d_i_ is the diameter of individual precipitated phase particles in the field of view, nm; A is the actual area of the field of view, nm2; t_v_ + d is the thickness of the sample, and t_v_ is taken as 100 nm; $\bar{d}$ is the average size of the precipitated phase in the field of view, nm. Substituting the average diameter of the precipitated phase in the different solid solution processes in the statistics into Equation (3), the volume fractions of precipitated phases of individual samples were calculated as shown in Table 7. From the table, it can be seen that the solid solution temperature has a great influence on the volume fraction of the precipitated phase; the volume fraction of the precipitated phase of the specimen held at 1040 °C for 5 min is more than 5 times that of the specimen held at 1080 °C for 5 min and is more than 30 times that of the specimen held at 1120 °C for 5 min.

**Figure 9:**
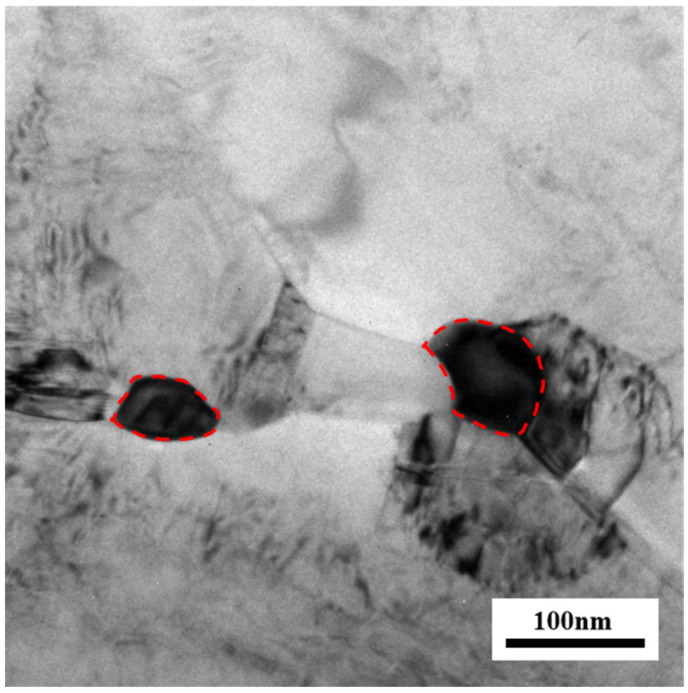
The relationship between precipitated phase and matrix. (Inside the dotted line is the precipitated phase).

**Figure 10:**
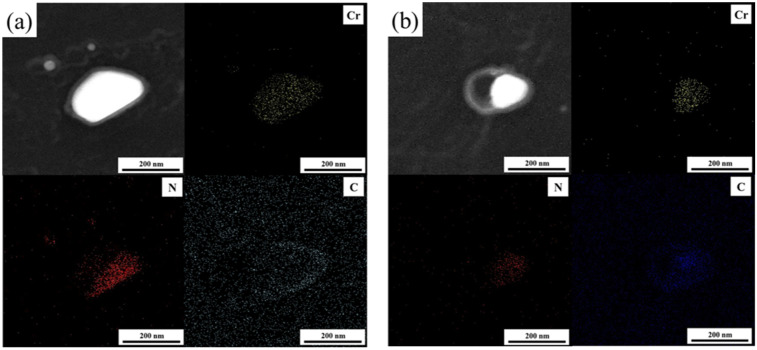
The distribution of Cr, N and C elements in the precipitated phase.

A certain magnification revealed that the precipitated phases in the test steels used in this study were mainly of spherical and ellipsoidal morphology. The precipitated phases are mainly precipitated along the grain boundary, as shown in Figure 9. To identify the types of precipitated phases, a transmission electron microscope (TEM) coupled with an energy-dispersive X-ray (EDX) spectrometer was utilized to perform surface scanning on various precipitated phases across random areas. Figure 10 shows the results of the EDS surface scan, the upper left is the dark field TEM image, and the rest is the distribution of the elements. From the results of the surface scan it can be seen that the precipitated phases are mainly chromium carbides and nitrides.

**Figure 11:**
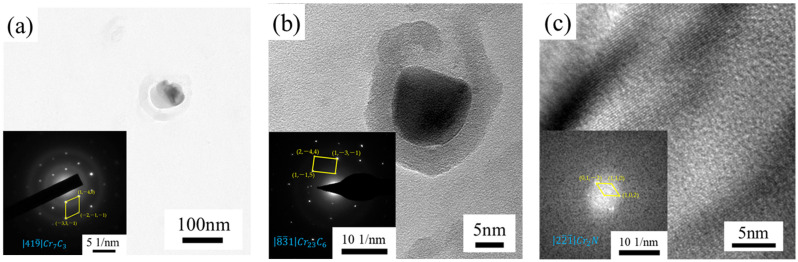
TEM image, SADP and diffraction pattern calibration of precipitated phase. (**a**) Cr_7_C_3_; (**b**) Cr_23_C_6_; (**c**) Cr_2_N.

Figure 11 shows the transmission electron micrographs of the precipitated phase in the test steel, the Selected Area Diffraction Pattern (SADP) of the precipitated phase and the calibration of the diffraction pattern. Among them, due to the small particle size of the precipitated phase in Figure 11c, the arrangement of atoms in the precipitated phase was obtained using high-resolution transmission electron microscopy (HRTEM) images, and then the HRTEM images were subjected to the DigitalMicrograph (Version 3.22.1461.0) software. Fast Fourier transform (FFT) was applied to the HRTEM images using DigitalMicrograph software to obtain the same images as the diffraction spots, and more information could be obtained by calibrating them to determine the type of the precipitated phase. In the lower left corner of each image, the diffraction spots of the precipitated phases are displayed along with their calibration results, confirming the presence of primarily Cr_7_C_3_, Cr_23_C_6_ and Cr_2_N. Cr_7_C_3_, a wear-resistant chromium carbide alloy developed by Beijing Naimo Company, boasts a surface hardness of HV1250. Cr_23_C_6_, with a density of 6.68 g/cm^3^ and a melting point of 1890 °C, is known for its excellent wear, corrosion and oxidation resistance at high temperatures. Cr_2_N is a nitride that precipitates at grain boundaries during heat treatment above 1210 °C and can transform into an ordered structure with increasing temperature. The reliability of the results was confirmed by comparison with the literature [33,34,35]. Combined with the surface scanning results of EDS, it can be determined that the precipitated phases of this steel grade are mainly of the Cr_x_C_y_ and Cr_x_N_y_ types after hot rolling and solid solution treatment. No Mo-rich precipitates have been detected in the EDS analysis and diffraction spot calibration, which should be related to the low content of Mo.

### 3.4. Strengthening Mechanism Analysis

It is generally accepted that the strengthening mechanisms of metallic materials can be grouped into four categories: grain boundary strengthening, precipitation strengthening, dislocation strengthening and solid solution strengthening. A large number of previous statistics and studies have pointed out that the overall contribution of different strengthening mechanisms to yield strength can be calculated by the Hall−Petch correction formula [32,36,37]. The calculation equation is as follows:

(4) $$\sigma_{yield}=\sigma_{0}+\sigma_{g}+\sigma_{p}+\sigma_{d}+\sigma_{s}$$

where $\sigma_{yield}$ is the theoretically calculated yield strength, MPa; $\sigma_{0}$ is the lattice friction stress, taken as 54 MPa [32]; $\sigma_{g}$ is the grain boundary strengthening increment, MPa; $\sigma_{p}$ is the precipitation strengthening increment, MPa; $\sigma_{d}$ is the dislocation strengthening increment, MPa; and $\sigma_{s}$ is the solid solution strengthening increment, MPa. 

Grain boundary strengthening is one of the important mechanisms to increase the yield strength of austenitic stainless steel. According to the Hall−Petch relationship [3], the yield strength increment caused by grain boundary strengthening is inversely proportional to the grain size as shown in Equation (5).

(5) $$\sigma_{g}=K\times \frac{1}{\sqrt{d}}$$

where K is the Hall−Petch coefficient and d is the average grain size.

For austenitic stainless steels, the Hall−Petch coefficient is typically 316.15 MPa μm^1/2^ [38]. From the above statistics for grain size, it can be seen that the average grain sizes of specimens #1, #2, #3, #4 and #5 are 11.42 μm, 12.70 μm, 13.37 μm, 13.90 μm and 15.12 μm, respectively, and therefore the amount of change in yield strength due to grain boundary strengthening is shown in Table 8.

Precipitation phases make an important contribution to the enhancement of material strength, which is mainly achieved by pinning dislocations and hindering dislocation movement. The precipitated phases, such as Cr_23_C_6_ and Cr_2_N in this study, can effectively inhibit dislocation slip through pinning, thus increasing the yield strength and tensile strength of the material. According to the Orowan strengthening mechanism, the increment $\sigma_{p}$ of precipitation relative to the yield strength can be calculated using Equation (6) [32].

(6) $$\sigma_{p}=\left(\frac{10.8\sqrt{V_{f}}}{d}\right)\ln\left(\frac{d}{6.125\times 10^{-4}}\right)$$

where V_f_ is the volume fraction of the precipitated phase and d is the average size of the precipitated phase particles in μm. For individual particles, larger particles (>60 nm) provide more strength than smaller particles. However, for a given volume fraction, the strengthening of the small particle number effect may outweigh the strengthening of particle size on the whole due to the greater number of small particles, so their overall contribution to strengthening may outweigh the effect of larger particles, which is why #1 is lower than #2 precipitation strengthening increments. Substituting the average diameter of the precipitated phase and the calculated volume fraction into Equation (6), the precipitation strengthening increment of each specimen was calculated, as shown in Table 9. 

Dislocations, a common type of line defect in metal materials, significantly impact the material’s organizational structure and mechanical properties. As they move, dislocations interact with neighboring ones, creating a strengthening effect. The higher the dislocation density, the more intense the interaction and the more pronounced the subsequent strengthening effect. The dislocation strengthening increment $\sigma_{d}$ of the test steel can be calculated by the Bailey−Hirsch formula [39], as shown in Equation (7).

(7) $$\sigma_{d}=M\alpha GB\rho^{\frac{1}{2}}$$

where M is the Taylor factor, taken as 3.06; α is the scale factor related to the crystal structure, taken as 0.38; G is the shear modulus, taken as 79 GPa for the austenitic matrix; b is the Burgers vector, taken as 0.248 nm for Fe; and ρ is the dislocation density, m^−2^. Substituting the dislocation density of each specimen in Table 6, the dislocation enhancement increment for each specimen was calculated as shown in Table 10.

The solid solution strengthening formula σs for experimental steels can be calculated using the following equation [40].

(8) $$\sigma_{s}=496\left[N\right]+356.5\left[C\right]+20.1\left[Si\right]+3.7\left[Cr\right]+14.6\left[Mo\right]$$

where [M] (M = C, Si, Mn…) is the content of elements solidly dissolved in the austenitic matrix, wt.%. According to Table 7, the volume fraction of precipitated phase under different solid solution processes is 0.077%, 0.051%, 0.023% and 0.013%, respectively. After calculation, if all elements in the precipitated phase are solidated, the strengthening increment on the matrix will not exceed 3.0 MPa at most. Therefore, it can be approximated that all elements are almost completely solidated in the matrix at normal temperature. Utilizing the mechanical performance calculation formula for non-quenched and tempered steels, the solid solution strengthening increment for each specimen was determined to be 185.99 MPa, as per Equation (8).

**Figure 12:**
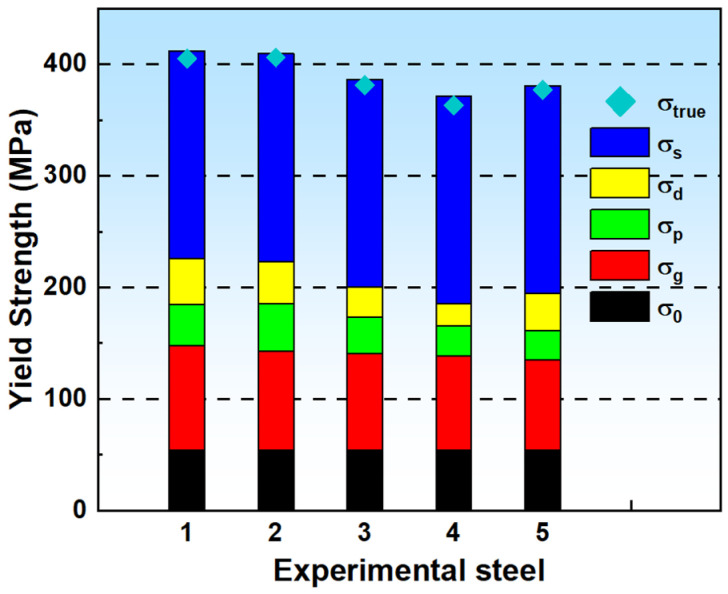
Incremental strengthening contributions from various mechanisms in experimental steels (MPa).

The strengthening increments of the above strengthening mechanisms are summarized and substituted into Equation (4) to calculate the theoretical yield strength of each experimental steel, and the results are shown in Figure 12 It can be seen that the calculated yield strengths of the experimental steels are very close to their measured yield strengths, with a difference of 15 MPa or less, indicating that the contribution of each strengthening mechanism to the yield strength obtained through the calculation has a certain degree of confidence, and the linear superposition of each strengthening increment is applicable to the current alloy system. Furthermore, upon comparing the contributions of various strengthening mechanisms, we identify solid solution strengthening and grain boundary strengthening as the primary mechanisms in the experimental steels. As the solid solution temperature rises from 1040 °C to 1080 °C, both grain boundary strengthening and dislocation strengthening in the experimental steels decrease, but the precipitates become smaller and more homogeneous, resulting in an increase in the precipitation strengthening, which is the reason why the experimental steels #1 and #2 have the same mechanical properties despite different conditions of solid solution treatment.

### 3.5. Fracture Mechanism Analysis

**Figure 13:**
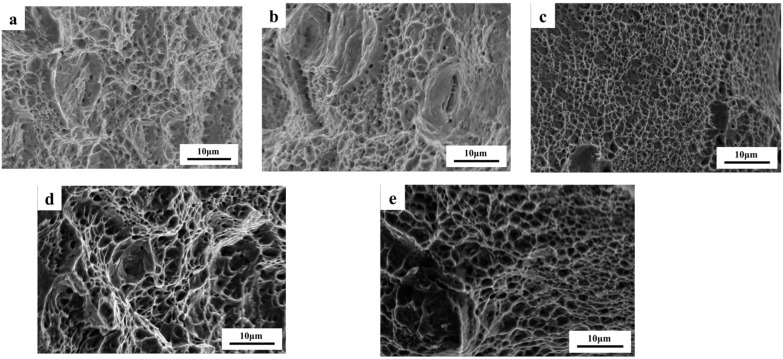
SEM fracture morphology of experimental steel: (**a**) 1040 °C, 5 min; (**b**) 1080 °C, 5 min; (**c**) 1080 °C, 15 min; (**d**) 1080 °C, 25 min; (**e**) 1120 °C, 5 min.

To conduct a more in-depth examination of the microstructural fracture mechanisms of experimental steel subjected to various solution conditions, a fracture analysis of tensile specimens was performed utilizing SEM. Figure 13a–e presents the fracture morphology of five experimental steels as observed through (SEM). The data indicate that experimental steels #1 and #2 predominantly exhibit ductile fracture characteristics, with some evidence of partial brittle fracture. In contrast, the remaining three experimental steels display typical ductile fracture features characterized by equiaxial dimples. As the temperature and duration of the solution treatment were elevated, there was a corresponding increase in both the size and depth of the dimple, suggesting an enhancement in the material’s plasticity. This observation aligns with the findings from the tensile tests. 

## 4. Conclusions

In this paper, the effect of the solution annealing process on the organization and properties of Cr−Mn−N system austenitic stainless steel was investigated, the strengthening mechanism was probed, and the following conclusions were drawn. 

(1) With the increase in solid solution annealing temperature and time, the grain size gradually increases. When the temperature reaches 1120 °C or the insulation at 1080 °C lasts for a longer period of time (25 min), the atomic diffusion rate in the austenite matrix accelerates, causing smaller grains to be gradually absorbed by the surrounding larger grains. This grain merging effect leads to a rapid increase in grain size and an increase in inhomogeneity.

(2) The TEM results show that there are more precipitated phases in the hot rolled specimens, and they are larger in size (200 nm), and the size of the precipitated phases is significantly reduced after the solid solution treatment. The volume fraction of the precipitated phase is significantly affected by the solid solution temperature, and the volume fraction of the precipitated phase in the sample held at 1040 °C for 5 min is more than 5 times that of the sample held at 1080 °C for 5 min and more than 30 times that of the sample held at 1120 °C for 5 min. Combined with EDS and HDTEM, it was determined that the precipitation phases of the steel grade after hot rolling and solution treatment were mainly Cr_7_C_3_, Cr_23_C_6_ and Cr_2_N phases.

(3) Solid solution strengthening and grain boundary strengthening are the main strengthening mechanisms of the steel. With the increase in the solid solution temperature and time, the increment of grain boundary strengthening and dislocation strengthening decreases, and the increment of precipitation strengthening increases and then decreases, which is the reason that the experimental steel with solid solution temperature of 1040 °C and solid solution temperature of 1080 °C held at 5 min still has the same mechanical properties despite the difference in solid solution treatment processes.

## Figures and Tables

**Table 1:** Chemical composition of experimental steel (wt.%).

Steel	C	N	Mn	Cr	Ni	Mo	P	S	Fe
201	0.14	0.14	9.22	13.25	1.02	0.003	0.038	0.003	Bal.

**Table 2:** Solid solution process of each specimen.

Specimens	Hot Rolled Specimen A	#1	#2	#3	#4	#5
Solid solution temperatures	/	1040 °C	1080 °C	1080 °C	1080 °C	1120 °C
Solid solution times	/	5 min	5 min	15 min	25 min	5 min

**Table 3:** Room temperature tensile properties of various samples.

Sample Number	#1	#2	#3	#4	#5
Yield strength/MPa	405	406	381	363	377
Tensile strength/MPa	975	971	951	931	958
Elongation at break/%	56.5%	56.0%	59.0%	60.0%	60.0%
Yield strength ratio	0.42	0.42	0.40	0.39	0.39

**Table 4:** Relative ratio and spanning ratio.

		Hot Rolled Specimen A	Solid Solution Treatment Samples				
			#1	#2	#3	#4	#5
relative ratio	(d_10(#)_ − d_10(original)_)/d_10(original)_	/	2.90	3.05	3.04	2.56	1.88
	(d_50(#)_ − d_50(original)_)/d_50(original)_	/	1.80	1.83	2.24	2.27	2.21
	(d_90(#)_ − d_90(original)_)/d_90(original)_	/	1.40	1.70	2.01	2.37	2.66
spanning ratio	(d_90_ − d_10_)/d_50_	1.98	1.52	1.42	1.73	2.02	2.35

**Table 5:** Average effective grain size.

Specimen Numbers	Hot Rolled Specimen	#1	#2	#3	#4	#5
Average effective Grain size/μm	4.42	11.29	12.58	13.33	13.81	15.06

**Table 6:** Geometrical dislocation density.

Specimen Numbers	Hot Rolled Specimen	#1	#2	#3	#4	#5
geometric dislocation density/×10^12^ m^−2^	11.8	3.67	3.05	1.55	0.80	2.39

**Table 7:** Volume fractions of precipitated phases under different solid solution processes.

Specimen Numbers	#1	#2	#3	#4	#5
f	0.401%	0.077%	0.051%	0.023%	0.013%

**Table 8:** Grain boundary strengthening increment under different solid solution processes.

Specimen Numbers	#1	#2	#3	#4	#5
$\sigma_{g}$/MPa	93.55	88.71	86.46	84.80	81.31

**Table 9:** Increment of precipitation strengthening under different solid solution processes.

Specimen Numbers	#1	#2	#3	#4	#5
$\sigma_{p}$/MPa	36.92	42.77	33.14	26.96	25.82

**Table 10:** Increment of dislocation strengthening under different solid solution processes.

Specimen Numbers	#1	#2	#3	#4	#5
$\sigma_{d}$/MPa	41.43	37.77	26.93	19.38	33.44

## Data Availability

The data presented in this study are available on request from the corresponding author due to the involvement of some confidential data from the company.
